# Evaluating the Safety and Efficacy of PD-1 Inhibitors in HIV Patients Diagnosed with Lung Cancer: A Systematic Review

**DOI:** 10.3390/ph18111654

**Published:** 2025-11-01

**Authors:** Helal F. Hetta, Yasser Alatawi, Fawaz E. Alanazi, Abdullah Alattar, Reem Alshaman, Hanan Alshareef, Zinab Alatawi, Majd S. Alatawi, Jumana H. Albalawi, Ghadeer A. Alosaimi, Reem Sayad, Wedad M. Nageeb

**Affiliations:** 1 Division of Microbiology, Immunology and Biotechnology, Department of Natural Products and Alternative Medicine, Faculty of Pharmacy, University of Tabuk, Tabuk 71491, Saudi Arabia; 2 Department of Pharmacy Practice, Faculty of Pharmacy, University of Tabuk, Tabuk 71491, Saudi Arabia; yasser@ut.edu.sa (Y.A.); halsharef@ut.edu.sa (H.A.); 3 Department of Pharmacology and Toxicology, Faculty of Pharmacy, University of Tabuk, Tabuk 71491, Saudi Arabia; falanazi@ut.edu.sa (F.E.A.); aalattar@ut.edu.sa (A.A.); ralshaman@ut.edu.sa (R.A.); 4 Department of Family and Community Medicine, Faculty of Medicine, University of Tabuk, Tabuk 47512, Saudi Arabia; zalatawi@ut.edu.sa; 5 PharmD Program, Faculty of Pharmacy, University of Tabuk, Tabuk 71491, Saudi Arabia; 451003956@stu.ut.edu.sa (M.S.A.); 421000067@stu.ut.edu.sa (J.H.A.); 6 Medical Laboratory Technology, College of Applied Medical Sciences, University of Tabuk, Tabuk 71491, Saudi Arabia; 421004661@stu.ut.edu.sa; 7 Department of Histology, Faculty of Medicine, Assiut University, Assiut 71515, Egypt; reem.17289806@med.aun.edu.eg; 8 Department of Medical Microbiology and Immunology, Faculty of Medicine, Suez Canal University, Ismailia 41522, Egypt; wedad_saleh@med.suez.edu.eg

**Keywords:** immune checkpoint inhibitors, programmed death-1, small-cell lung cancer, non-small-cell lung cancer, immunodeficiency disease

## Abstract

**Background and Aim**: People with HIV (PWH) have historically been excluded from cancer immunotherapy trials due to concerns over immune dysregulation and safety. This systematic review evaluates the safety, efficacy, and immunologic outcomes of Programmed death-1 (PD-1) inhibitors in PWH diagnosed with non-small-cell lung cancer (NSCLC). **Methods**: Following PRISMA guidelines, a systematic search was conducted across PubMed, Scopus, Web of Science, and Medline through January 2025. Studies were included if they reported outcomes of ICIs in PWH with NSCLC. Data extraction included progression-free survival (PFS), overall survival (OS), immune-related adverse events (irAEs), antitumor response, HIV viral control, and immunologic parameters. Study quality was assessed using the Joanna Briggs Institute (JBI) checklist. **Results**: Six cohort studies (*n* = 762 patients) met inclusion criteria. ICIs used included nivolumab, pembrolizumab, atezolizumab, and durvalumab, with treatment durations ranging from 3.1 to 5.4 months. Median PFS ranged from 3.0 to 6.3 months, and OS ranged from 10.0 to 66.0 months. Overall response rates (ORRs) varied from 13% to 75%, and disease control rates (DCRs) ranged from 47% to 62.5%. irAEs occurred in 25% to 75% of patients, with 6–20% experiencing grade 3–4 events. Corticosteroids were required in 13–29% of patients, and treatment discontinuation due to toxicity occurred in up to 30%. Most patients had controlled HIV, with CD4 counts typically above 300 cells/μL and undetectable viral loads. **Conclusions**: ICIs appear safe and effective in PWH with NSCLC, with toxicity and efficacy outcomes comparable to the general population. While immunotherapy should not be withheld based solely on HIV status, better standardization in reporting HIV-related variables is needed to optimize patient selection and management.

## 1. Introduction

Antiretroviral therapy (ART) has made significant strides in the last 20 years, bringing human immunodeficiency virus (HIV) replication and its fatal effects under consistent control. Because of this, people with HIV infection (PWH) are living longer and are more likely to develop a new cancer as they get older [1]. The incidence of these cancers is higher in PWH than in HIV-uninfected people [2]. T-cell-mediated immune responses against different cancer types are restored by immune checkpoint inhibitors (ICIs), such as monoclonal antibodies that block cytotoxic T-lymphocyte-associated protein 4 (CTLA-4), programmed death 1 (PD-1), or its ligand (PD-L1) [3].

PWH have an approximately 2–4-fold higher incidence of lung cancer than the general population, even after controlling for smoking and other confounders [4,5]. This elevated risk is multifactorial, reflecting the combined effects of chronic immune activation, impaired immunosurveillance, persistent pulmonary inflammation, and co-infections such as *Pneumocystis jirovecii* or tuberculosis that may contribute to lung tissue damage and carcinogenesis. Furthermore, prolonged exposure to oxidative stress and ART-related metabolic changes may also promote oncogenic transformation. Given these mechanisms, HIV-associated non-small-cell lung cancer (NSCLC) represents a biologically distinct and clinically challenging entity, with implications for both tumor immunogenicity and responsiveness to immune checkpoint blockade.

Over time, PWH experience chronic immune activation and systemic inflammation despite effective viral suppression with ART. This persistent inflammatory milieu drives metabolic and signaling alterations that predispose to oncogenesis. Continuous activation of the PI3K/AKT/mTOR and NF-κB pathways promotes cell survival, proliferation, and resistance to apoptosis, while JAK/STAT signaling contributes to dysregulated cytokine release and angiogenesis. HIV proteins such as Tat and Nef further induce oxidative stress, DNA damage, and epigenetic dysregulation, amplifying genomic instability. In parallel, long-term ART exposure can cause mitochondrial dysfunction, lipid metabolism alterations, and insulin resistance, creating a pro-oncogenic metabolic environment. Combined with reduced CD4^+^ T-cell-mediated immune surveillance, these mechanisms facilitate malignant transformation and progression of non-AIDS-defining cancers such as NSCLC in PWH [6,7,8].

Beyond lung cancer, HIV infection significantly increases the risk of several malignancies. AIDS-defining cancers (ADCs) include Kaposi’s sarcoma, non-Hodgkin lymphoma, and invasive cervical cancer, which were historically the most frequent in untreated HIV. However, with widespread use of combination ART, the incidence of ADCs has declined. At the same time, non-AIDS-defining cancers (NADCs), such as Hodgkin lymphoma, hepatocellular carcinoma, anal cancer, head and neck squamous cell carcinoma, and lung cancer, have become more prevalent. This shift reflects improved immune restoration and longevity among people with HIV, alongside persistent chronic inflammation, viral co-infections, and metabolic and immune dysregulation that continue to promote oncogenesis [2,9].

ICIs do not seem to interact with ART and are quite successful in reestablishing strong antitumor immunity [10]. They are currently often used to treat a variety of cancers since their use has significantly increased the survival rate of cancer patients [11]. Nevertheless, ICIs may potentially impair immunological tolerance, increasing the likelihood of autoimmune responses and other immune-related adverse events (irAEs) [12]. PWH were initially excluded from all oncology trials that assessed the safety and effectiveness of ICIs [13]. PWH are nevertheless more susceptible to infections, malignancies, and inflammatory symptoms, even with the high efficacy of ARTs [14,15,16,17]. Therefore, it was believed that using ICIs in PWH might result in a larger rate of irAEs, a reduced antitumoral activity, and/or the production of inflammatory syndromes [18,19,20]. Reassuring information about their use in this population with a unique immunological profile has surfaced more recently. A 12.1% rate of severe adverse events (AEs) (Common Terminology Criteria for Adverse Events (CTCAE) grade > 2) was found in 176 PWH treated with ICIs, mostly from retrospective studies. This rate is similar to the general population [3].

Three phase I/II trials that examined the use of durvalumab, nivolumab, and pembrolizumab in virally controlled PWH with cancer supported this conclusion. The trials found that the rates of severe AEs were 0%, 6.3%, and 23.3%, respectively [21,22,23].

Although it is still a rare occurrence to date, it is noteworthy that one participant who received pembrolizumab for Kaposi sarcoma-associated herpes virus infection (KSHV) experienced a fatal polyclonal KSHV-associated B-cell lymphoproliferation [23]. The results were similarly encouraging regarding the impact of ICIs on HIV viral indicators, including circulating CD4 T levels and plasma viral load (VL). In the systematic study, 61% of people receiving therapy had consistent circulating CD4 T levels (within ±100 CD4/mm^3^), and 92% of participants had a stable VL (within ±50 copies/mL) [3].

There is currently little information available on the anticancer effectiveness of ICIs in PWH. The three-phase I/II trials only involved a small number of people; however, they indicated efficacy comparable to the general population [21,22,23]. Furthermore, a recent large control-matched retrospective analysis did not find any difference between immunocompetent participants with metastatic NSCLC and PWH with metastatic NSCLC in terms of overall survival (OS) or progression-free survival (PFS) [24].

There are arguments in favor of immunotherapy having a possibly positive impact on HIV infection itself, in contrast to these possible negative effects. Indeed, inhibiting the PD-1/PD-L1 axis would enhance HIV-specific CD4 and CD8 T cell functions [25,26], which may have an impact on HIV reservoirs, because PD-1 is overexpressed on HIV-specific CD4 and CD8.

Therefore, this systematic review aims to evaluate the safety and efficacy of ICIs in PWH diagnosed with lung cancer, including NSCLC and small-cell lung cancer (SCLC). Specifically, we assess clinical outcomes such as prognosis, PFS, and OS, as well as irAEs and treatment-related immune parameters.

## 2. Methods

### 2.1. Information Sources and Search Strategy

The Preferred Reporting Items for Systematic Reviews and Meta-Analyses (PRISMA) were taken into consideration when creating a systematic review of clinical trials [27]. The registration number for this systematic review on PROSPERO is CRD420251058709. The Web of Science (WoS), Medline, PubMed, SCOPUS, and WoS databases were analyzed through January 2025. Immunocheckpoints that act as a checkpoint on the immune system, including receptor programmed death 1 (PD-1), are blocked by monoclonal antibodies known as ICIs. This improves the immune response to a tumor. Furthermore, we covered any HIV-related lung cancer, including NSCLC and SCLC. The cohorts published up until January 2025 were reviewed using these parameters. Next, we searched every database using the Boolean operators AND and OR. The search strategy’s specifics include: (Prolgolimab OR Nivolumab OR Pembrolizumab OR Atezolizumab OR Durvalumab OR Avelumab OR Cemiplimab OR Tislelizumab OR Dostarlimab OR Retifanlimab OR Programmed cell death protein 1 OR PD-1 Protein OR Programmed Cell Death 1 Receptor OR “Immune checkpoint inhibitors” OR “PD-1 inhibitors” OR “PD-L1 inhibitors” OR “CTLA-4 inhibitors” OR “checkpoint blockade therapy” OR “immune checkpoint blockade” OR “checkpoint inhibitors” OR “ipilimumab”) AND (“cancer” OR “neoplasm” OR “malignancy” OR “solid tumors” OR “carcinoma” OR “oncology” OR “NSCLC” OR “non-small cell lung cancer” OR “non small cell lung cancer”) AND (“HIV” OR “Human Immunodeficiency Virus” OR “HIV-positive” OR “AIDS” OR PLWH OR MHIV OR WHIV OR “HIV-infected” OR “HIV-associated malignancies” OR “AIDS-related cancer”).

### 2.2. Eligibility Criteria

Inclusion criteria: All eligible papers found for inclusion reported results exclusively for NSCLC, even though both NSCLC and SCLC were included in the original search strategy. There were no studies that specifically examined SCLC in HIV patients receiving ICIs that met the inclusion criteria. Criteria for exclusion: In vitro experiments, animal research, case reports, case series, and reviews were not accepted.

### 2.3. Research Questions

The following question is the focus of this systematic review. (a) What is the impact of immune checkpoint inhibitors on prognosis, PFS, and OS as the main outcomes in patients with HIV who have lung cancer? (b) What are irAEs of immune checkpoint inhibitors? (c) How many lung cancer patients who receive immune checkpoint inhibitors survive? This survive?

### 2.4. Trials Selection

After reading the abstracts and full texts, certain keywords prompted both researchers to choose the papers. The two researchers used the inclusion criteria to assess the studies. Subsequently, every abstract and full text was downloaded and evaluated independently based on the pre-established inclusion criteria. When there was disagreement among the authors, the third author assessed the acceptability of the study.

### 2.5. Data Extraction

Two authors independently reviewed and evaluated each full text that met the inclusion criteria to be included in this systematic review. Each investigator independently created a table that included the most crucial details from the chosen trials, and the outcomes were compared.

### 2.6. Outcome Measures

The primary outcomes are (a) the efficacy of immune checkpoint inhibitors on prognosis, PFS, and OS. Secondary outcomes are (b) the immune checkpoint inhibitors’ irAEs and (c) the survival rate of lung cancer patients receiving ICIs. (d) The prognosis was presented in the forms of complete remission (CR), partial remission (PR), stable disease (SD), progressive disease (PD), PFS, and OS.

### 2.7. Quality Assessment

To evaluate the methodological quality of the included case series, we used a standardized 10-item critical appraisal checklist based on the Joanna Briggs Institute (JBI) tool [28]. Each study was assessed across ten domains, including clarity of inclusion criteria, standard and valid methods of condition identification, participant selection (consecutive and complete inclusion), reporting of demographics and clinical data, outcome reporting, and appropriateness of statistical analysis. Each item was rated as “Yes”, “No”, or “Not clear” based on the information provided in the published articles.

### 2.8. Data Analysis

For each included study, we extracted and summarized outcome data related to PFS, OS, irAEs, treatment-related interventions, HIV-related immunologic parameters, and antitumor response rates in patients with NSCLC. Data was stratified by study and assessed at the latest available time points reported where available.

For time-to-event data (PFS and OS), we recorded the reported mean or median durations along with corresponding 95% confidence intervals (CIs). Categorical outcomes—including all-grade and grade-specific irAEs (grades 1–2 and 3–4), mortality rates, requirement for corticosteroid therapy, and treatment discontinuation due to toxicity—were summarized as proportions (event counts over total evaluable patients) for each study cohort.

Descriptive statistics were used to summarize continuous variables, including CD4 count, CD4 nadir, and CD4:CD8 ratio. These were expressed as means with standard deviation (SD) or medians with interquartile ranges (IQR), depending on how they were reported in the source studies. HIV RNA viral load suppression (e.g., proportion of patients with undetectable viremia) and median viral loads among viremic patients were also summarized when available.

Antitumor response was evaluated based on overall response rate (ORR) and disease control rate (DCR), as defined by each study’s criteria (typically RECIST 1.1). ORR was defined as the sum of complete and partial responses, while DCR additionally included patients with stable disease. Where provided, 95% CIs were reported for response rates.

Due to substantial heterogeneity in study design (prospective vs. retrospective cohorts), patient characteristics, and outcome reporting formats, a quantitative meta-analysis was deemed inappropriate. The included studies varied in their reporting of PFS, OS, and irAEs, which were presented using different statistical measures and follow-up intervals. Moreover, HIV-specific parameters—such as CD4 count, viral load control, and antiretroviral therapy status, were inconsistently described across studies. In accordance with PRISMA guidelines, we therefore conducted a structured descriptive synthesis, tabulating and narratively comparing safety and efficacy outcomes across the included cohorts rather than performing meta-analytic pooling.

## 3. Results

### 3.1. Study Selection

**Figure 1:**
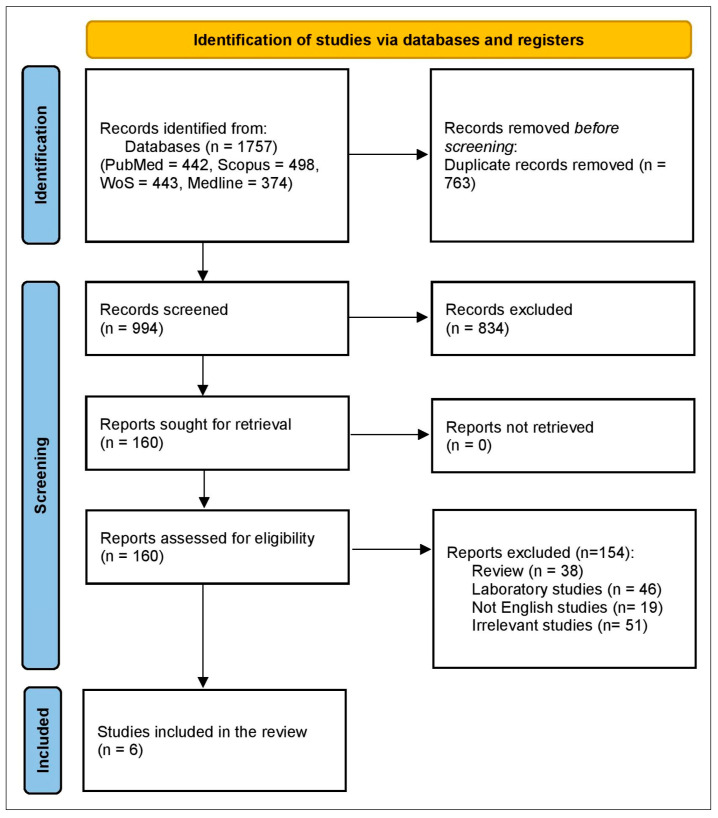
PRISMA flowchart.

Initial retrieval of 1757 studies, with 763 excluded as duplicates, leaving 994 for title and abstract screening. From these, 834 were excluded after title and abstract screening, leaving 160 studies for full-text screening. Finally, 154 studies were excluded, resulting in six cohort studies (Figure 1).

### 3.2. Study Characteristics

All six eligible studies focused on NSCLC populations; no studies reporting on SCLC met the inclusion criteria, comprising prospective and retrospective cohort designs and a phase 2 clinical trial, published between 2018 and 2024 [22,29,30,31,32,33]. The studies assessed the safety and efficacy of PD-1/PD-L1 ICIs in PWH diagnosed with NSCLC. The total sample size across studies was 762 patients, with individual study populations ranging from 16 to 492 participants. Median age ranged from 58 to 69 years, and where reported, most patients were male.

All included studies involved the administration of PD-1 or PD-L1 inhibitors—most commonly nivolumab, pembrolizumab, atezolizumab, and durvalumab. In one study, combinations with anti-CTLA-4 (ipilimumab) and anti-VEGFR (bevacizumab) were also used [29]. The median duration of ICIs therapy ranged from 3.1 to 5.4 months, with treatment typically initiated in the second or third line. Only a few studies specified the lines of prior therapy [22,33].

About HIV-related variables, all studies reported that the majority of PWH were on ART at baseline. Three studies explicitly stated that participants had controlled HIV infection (e.g., viral load <200 copies/mL), while others either did not report viral control or AIDS status in detail [22,30,33]. In the Leonardi et al. cohort, 80% were asymptomatic for AIDS, and 20% were receiving immunosuppressive or immunomodulatory treatment for AIDS at enrollment [33].

A history of autoimmune diseases was variably reported. Assoumou et al. and Leonardi et al. documented a wide range of autoimmune conditions among participants, including rheumatoid arthritis, psoriasis, Crohn’s disease, and myositis [29,33]. Leonardi et al. noted that 20% of participants were on active treatment for autoimmune diseases at the time of ICIs initiation, including corticosteroids and steroid-sparing agents [33]. Cortellini et al. identified various immune-related adverse events across several organ systems but did not quantify baseline autoimmune comorbidities [31].

Smoking history was inconsistently reported. Only Leonardi et al. provided detailed data, noting that 88% were former smokers, 7% never smoked, and 5% were current smokers [33]. Overall, the use of ICIs in PWH with NSCLC appeared to be feasible, with many patients able to continue ART throughout cancer treatment. However, variability in reporting, particularly on AIDS symptomatology, HIV viral control, and autoimmune disease management, limits the generalizability of findings. Table 1 presents more details about the baseline characteristics of the patients included.

### 3.3. Efficacy Outcomes

Across the included studies evaluating ICIs in patients with NSCLC and HIV, PFS ranged from a median of 3.0 to 6.3 months, with longer PFS observed at earlier timepoints in Assoumou et al., where median PFS was 48.8 months at 6 months, 32.3 months at 12 months, and 25.5 months at 18 months [29]. OS was variably reported, with Assoumou et al. estimating OS to be 66.0 months at 6 months, declining to 45.1 and 36.4 months at 12 and 18 months, respectively [29]. Other studies reported shorter median OS, such as 16.0 months in El Zarif et al. [30], 10.0 months in Cortellini et al. [31], and 10.9 months in Lavole et al. [22].

Mortality was reported in several studies, with the proportion of patients who died ranging from 47.7% in El Zarif et al. [30] to 77% in Cortellini et al. [31]. The incidence of any irAEs varied across studies, ranging from 25% [32] to 75% [22]. Grade 1–2 irAEs were more common than grade 3–4 irAEs, with the latter occurring in 6–20% of patients when reported. The use of corticosteroids for irAE management was reported in 13–29% of patients across studies, with 14–30% requiring temporary or permanent discontinuation of ICIs therapy due to toxicity.

Immunologic and virologic characteristics of the HIV-positive populations were inconsistently reported. Assoumou et al. and Cortellini et al. both described preserved immune function, with median CD4 counts of 336 (IQR: 210–598) and 491 (SD: ± 182) cells/µL, and 84–100% of patients having undetectable HIV viral loads [29,31]. Similar findings were reported by El Zarif et al. and Shah et al. [30,32], although details were less complete.

Response rates to ICIs were heterogeneous across studies. The ORR ranged from 13% (Shah et al., monotherapy subgroup) to 75% (Shah et al., ICIs + chemotherapy) [32]. Other reported ORRs included 22% [29,33], 20% [31], and 31% [30]. DCR ranged from 47% to 62.5%, where available. Notably, the response assessments varied slightly in methodology and follow-up time across studies. More details of the prognosis of the disease are presented in Table 2.

### 3.4. Quality Assessment Results

All six included studies met the majority of the quality criteria for case series. Five of the six studies reported appropriate and valid methods for identifying the condition, consistent and reliable measurement of outcomes, and complete inclusion of participants [22,30,31,32,33]. Additionally, all studies provided clear reporting of clinical characteristics and demographic data. Four studies had clearly defined inclusion criteria [30,31,32,33], while two lacked clarity on how participants were selected into the cohort [22,29]. Nonetheless, both still adhered to consistent methods for measuring and identifying the condition and met other key appraisal criteria (Table 3).

All studies conducted appropriate statistical analyses and reported outcomes or follow-up findings. Only one study presented some aggregated data not specific to NSCLC or HIV subgroups, which may limit interpretability in this subgroup analysis [31].

**Table 1:** Summary of the included studies and baseline characteristics of the patients.

Study (Year)	Design/ Duration	Sample Size (*n*)	Median Age (Years)	Male, *n* (%)	Cancer Type/Stage	PD-1/PD-L1 Inhibitor(s)	Combination or Prior Therapy	Median Treatment Duration (Months)	ART at Baseline	HIV/AIDS Status	Autoimmune Disease (If Any)
Assoumou et al., 2024 [29]	Prospective observational cohort (Jan 2018–Dec 2023)	65	59 (IQR 54–64)	48 (74%)	Lung cancer (mixed histology, stage not specified)	Pembrolizumab, nivolumab, cemiplimab, atezolizumab, durvalumab; ± ipilimumab or bevacizumab	Prior chemo/radiotherapy/targeted therapy in some patients	5.4 (IQR 2.1–12.7)	All on ART	Majority asymptomatic; AIDS data not detailed	Autoimmune hemolytic anemia, Crohn’s disease, myositis, rheumatoid arthritis, psoriasis, ITP, ankylosing spondylitis
El Zarif et al., 2023 [30]	Retrospective multicenter cohort (2015–2021)	111	58 (51–63)	Not reported	NSCLC (mixed histology/stage)	Nivolumab, pembrolizumab, atezolizumab, durvalumab	Prior chemotherapy, TKIs, antiangiogenic therapy, or dual ICIs	Not reported	All on ART	AIDS status not reported	Not reported
Lavole et al., 2021 [22]	Nonrandomized, open-label phase 2 trial (2017–2019)	16	58 (44–71)	14 (88%)	NSCLC: adenocarcinoma 63%, squamous 31%; stage IIIB–IVB	Nivolumab 3 mg/kg IV q2wk	None	3.5 (0.5–26.5)	All on cART	All with controlled HIV (VL < 200 copies/mL)	Not reported
Cortellini et al., 2019 [31]	Retrospective (2013–2018)	492	69 (24–92)	Not reported	NSCLC (advanced)	Pembrolizumab, nivolumab	None	Not reported	Not reported	HIV+ subgroup; AIDS data not detailed	Thyroid, dermatologic, rheumatologic, GI/hepatic, neurologic disorders
Shah et al., 2019 [32]	Retrospective (2011–2018)	22	62 (29–85)	Not reported	NSCLC (mixed)	Nivolumab, pembrolizumab, atezolizumab, durvalumab, avelumab	None	Not reported	Most on ART (various regimens)	Not reported	Not reported
Leonardi et al., 2018 [33]	Retrospective cohort (2015–2017)	56	67 (45–90)	21 (38%)	NSCLC: adenocarcinoma 73%, squamous 25%; stage IIIB–IV	Nivolumab (80%), pembrolizumab (18%), atezolizumab (2%)	None	3.1 (95% CI 1.8–5.1)	Majority on ART	20% symptomatic AIDS; 11 receiving immunosuppressive therapy	Rheumatologic (45%), dermatologic (29%), endocrine (16%), GI (11%), neurologic (5%)

Abbreviations: ART = antiretroviral therapy; AIDS = acquired immunodeficiency syndrome; cART = combined antiretroviral therapy; ICIs = immune checkpoint inhibitors; ITP = immune thrombocytopenic purpura; NSCLC = non-small-cell lung cancer; PD-1 = programmed death-1; PD-L1 = programmed death ligand-1; TKI = tyrosine kinase inhibitor; VL = viral load.

**Table 2:** Summary of outcomes of the included studies.

Study (Year)	Point of Assessment	Progression-Free Survival (Months)	Overall Survival (Months)	Deaths *n* (%)	Any irAE *n* (%)	Grade 1–2 irAEs *n* (%)	Grade 3–4 irAEs *n* (%)	Treatment Required for irAEs *n* (%)	CD4 Count (Cells/µL)	CD4 Nadir (Cells/µL)	CD4:CD8 Ratio	HIV RNA (Copies/mL)	Response (ORR/DCR)
Assoumou et al., 2024 [29]	6, 12, 18 months	6 mo: 48.8 (40.1–57.0); 12 mo: 32.3 (24.3–40.5); 18 mo: 25.5 (17.8–33.5)	6 mo: 66.0 (52.8–76.4); 12 mo: 45.1 (31.8–57.6); 18 mo: 36.4 (23.7–49.3)	81/140 (58%)	Not available	Not available	20 (15.0% at 12 mo; 18.7% at 18 mo)	41/140 (29.3%) received glucocorticoids	336 (210–598)	117 (51–240)	0.7 (0.3–1.0)	VL < 50 in 84%; viremic median 460 (IQR: 106–39,550)	ORR 22% (PR 11/50); DCR 53% (SD 15/50)
El Zarif et al., 2023 [30]	Not reported	6.3 (4.3–10.1)	16.0 (10.6–40.2)	53/111 (47.7%)	12 (20%)	Not reported	7 (12%)	6/61 (9.8%)	314 (206–472)	Not available	Not available	VL <400 in 96%	ORR 31% (32/102); 95% CI 23–41%
Lavole et al., 2021 [22]	Median follow-up: 23.6 mo	3.4 (1.8–5.6)	10.9 (2.2–NR)	Not stated (based on 10 patients)	12 (75%)	11 (69%)	1 (6%)	One serious AE, managed clinically	385 (187–778)	274 (32–778)	Not reported	All controlled (VL < 50)	DCR 62.5% (PR 2, SD 8, PD 5)
Cortellini et al., 2019 [31]	Not reported	3.0 (2.0–4.1)	10.0 (6.6–13.4)	23/30 (77%)	16 (53%)	10 (33%)	6 (20%)	6 (20%) received corticosteroids; 9 (30%) discontinued ICIs	491 ±182	196 ±125	0.7 ±0.3	All undetectable (<50 copies/mL)	ORR 20%; DCR 47%
Shah et al., 2019 [32]	Not reported	Not available	Not available	Not available	3 (25%)	1 (8%)—rash (ICIs + chemo)	2 (17%)—pneumonitis (ICIs mono)	Not reported	Not reported	Not reported	Not reported	4/6 undetectable VL pre-ICIs	ICIs monotherapy ORR 13% (1 CR); ICIs + chemo ORR 75% (3 PR)
Leonardi et al., 2018 [33]	Not reported	3.1 (1.8–5.1)	Not available	Not available	22 (38%)	15 (27%)	6 (11%)	7 (13%) corticosteroids; 14% discontinued ICIs	Not reported	Not reported	Not reported	Not reported	ORR 22% (11 PRs); DCR 53%

Abbreviations: AE = adverse event; CD = cluster of differentiation; CI = confidence interval; CR = complete response; DCR = disease control rate; HIV = human immunodeficiency virus; ICIs = immune checkpoint inhibitors; irAE = immune-related adverse event; IQR = interquartile range; ORR = overall response rate; PD = progressive disease PR = partial response; SD = stable disease; VL = viral load.

**Table 3:** Quality assessment of the included studies. * Most information represents the whole study, not specific to NSCLC.

Study ID	Leonardi et al., 2018 [33]	Cortellini et al., 2019 * [31]	Shah et al. (2019) [32]	Lavole et al., 2021 [22]	El Zarif et al., 2023 [30]	Assoumou et al., 2024 [29]
**Were There Clear Criteria for Inclusion in the Case Series?**	Yes	Yes	Not clear	Not clear	Yes	Not clear
**Was the Condition Measured in a Standard, Reliable Way for All Participants Included in the Case Series?**	Yes	Yes	Yes	Yes	Yes	Yes
**Were Valid Methods Used for Identification of the Condition for all Participants Included in the Case Series?**	Yes	Yes	Yes	Yes	Yes	Yes
**Did the Case Series Have Consecutive Inclusion of Participants?**	Yes	Yes	Yes	Yes	Yes	Yes
**Did the Case Series Have Complete Inclusion of Participants?**	Yes	Yes	Yes	Yes	Yes	Yes
**Was There Clear Reporting of the Demographics of the Participants in the Study?**	Yes	Yes	Yes	Yes	Yes	Yes
**Was There Clear Reporting of Clinical Information of the Participants?**	Yes	Yes	Yes	Yes	Yes	Yes
**Were the Outcomes or Follow-up Results of Cases Clearly Reported?**	Yes	Yes	Yes	Yes	Yes	Yes
**Was There Clear Reporting of the Demographics of the Participants in the Study?**	Yes	Yes	Yes	Yes	Yes	Yes
**Was Statistical Analysis Appropriate?**	Yes	Yes	Yes	Yes	Yes	Yes

Overall, the methodological quality of the included studies was high, although some studies lacked clarity in reporting inclusion criteria, which may introduce a risk of selection bias.

## 4. Discussion

This systematic review synthesized data from six cohort studies evaluating the safety and efficacy of PD-1/PD-L1 ICIs in PWH diagnosed with NSCLC. Despite historical exclusion of this population from clinical trials, the findings suggest that ICIs therapy is both feasible and clinically meaningful for PWH, with manageable safety profiles and evidence of antitumor activity.

Across the included studies, efficacy outcomes showed variable but encouraging results. Reported median PFS ranged from 3.0 to 6.3 months, aligning with those observed in the general NSCLC population. Assoumou et al. notably reported longer PFS and OS at interim time points, though these findings were not consistently replicated in other cohorts [29]. Median OS in the remaining studies ranged from approximately 10 to 16 months. These data indicate that, when appropriately selected, PWH with NSCLC may derive comparable survival benefits from ICIs as their HIV-negative counterparts.

Beyond clinical and immunologic factors, underlying genetic alterations may also play an essential role in shaping responses to PD-1/PD-L1 inhibitors. Mutations in TP53 and KRAS, frequently observed in NSCLC, are associated with enhanced tumor mutational burden, increased PD-L1 expression, and altered immune microenvironment, potentially contributing to improved responsiveness to ICIs. Conversely, specific oncogenic pathways may promote immune evasion through modulation of antigen presentation and interferon signaling. While the included studies did not report molecular profiles, integrating genomic biomarkers into future analyses of HIV-associated NSCLC could provide critical mechanistic insight and refine therapeutic decision-making for this unique patient population [34,35].

Historically, platinum-based chemotherapy represented the mainstay of treatment for HIV-positive patients with advanced NSCLC, with reported median overall survival ranging between 6 and 10 months and substantial hematologic and infectious toxicities. In contrast, contemporary real-world studies of PD-1/PD-L1 inhibitors in this population have demonstrated median overall survival of approximately 10–16 months, with response rates (ORR 20–30%) comparable to those observed in HIV-negative cohorts [36,37,38,39,40]. Importantly, irAEs were generally manageable and did not result in opportunistic infections or HIV reactivation. These findings suggest that immune checkpoint blockade may offer at least equivalent efficacy and improved tolerability relative to conventional chemotherapy, particularly in patients receiving effective ART with immune reconstitution.

Although ART remains essential for viral suppression and immune restoration, its long-term use is associated with several toxic side effects. These include hepatotoxicity, nephrotoxicity, dyslipidemia, insulin resistance, mitochondrial dysfunction, and cardiovascular complications, mainly resulting from nucleoside reverse transcriptase inhibitors (NRTIs) and protease inhibitors (PIs). Additionally, chronic inflammation and oxidative stress linked to ART exposure may contribute to endothelial damage and metabolic abnormalities. In cancer patients, these toxicities can overlap with those from immunotherapy or chemotherapy, potentially increasing the risk of hepatic or metabolic adverse events. Therefore, individualized ART selection, close toxicity monitoring, and coordination between oncologists and infectious disease specialists are essential to ensure safety and optimize therapeutic outcomes [41,42].

The safety profile of ICIs in this population was also reassuring. The incidence of irAEs ranged from 25% to 75%, with most being grade 1–2. Severe (grade 3–4) irAEs occurred in 6–20% of cases and were typically manageable with corticosteroids. Treatment discontinuation due to toxicity occurred in a minority of patients (14–30%), reflecting known risks of immunotherapy use and not exceeding expected rates in the broader NSCLC population. These findings support the growing consensus that PWH can safely receive ICIs therapy under appropriate monitoring.

The ANRS CO24 OncoVIHAC cohort study conducted in France has prompted the deployment through a prospective, real-world national cohort to assess the safety of PWH receiving ICIs for various cancer types in PWH. This is due to the close collaboration between oncologists, infectiologists, and pharmacologists that was established through the ONCOVIH network within the framework of regular national or regional interdisciplinary meetings. 13.8% (95% CI: 8.8% to 21.4%) of PWH experienced a severe adverse event at 6 months, 15.0% (95% CI: 9.6% to 22.9%) at 12 months, and 18.7% (95% CI: 12.1% to 28.3%) at 18 months, according to their findings over a 2-year follow-up [29]. As most events were reversible following systemic glucocorticoid use and subsequently safely managed, this is consistent with the results of irAEs in HIV-uninfected populations, showing that such therapies can be used in PWH who did not experience excessive treatment-related immune toxicities. Between 5% and 30% of individuals in the general community experience grade ≥3 irAEs [43]. Furthermore, grade ≥3 irAEs were 15.1% for atezolizumab, 14.1% for nivolumab, 19.8% for pembrolizumab, and 28.6% for ipilimumab, according to a systematic review and meta-analysis of data from 36 phase II and III randomized controlled trials (*n* = 15,370) involving HIV-uninfected individuals with cancer [44]. In contrast to their findings, the study conducted in PWH by El Zarif et al. revealed a comparatively lower incidence rate of grade ≥3 irAEs at 6 months, 7.7% [30]. This discrepancy might be explained by the first study’s retrospective design, which led to selection bias and the exclusion of some events. According to Wang et al., the incidence of fatal ICIs-associated AEs was 0.7%, which is comparable to the 0.3% to 1.3% observed in HIV-uninfected people [45]. Another study found that nivolumab had a lower incidence of irAEs than other ICIs [46]. Furthermore, viral infections like Cytomegalovirus (CMV) may contribute to the remodeling of the immunological milieu surrounding the tumor, changing the host immune response and hence promoting the occurrence of irAEs [47]. Surgery is likely to cause irAEs since it can also result in immunogenic lesions on non-malignant host cells. A longer time after HIV diagnosis and a lower CD4 cell count were associated with a higher risk of severe treatment-related toxicity.

The immunologic status of patients, as reported, further supports the use of ICIs in this setting. Most patients had well-controlled HIV, with high rates of ART adherence and suppressed viral loads. CD4 counts were generally preserved, and the few studies reporting CD4:CD8 ratios or nadir values described ranges consistent with stable immune function. However, the limited and inconsistent reporting of HIV-related parameters—including AIDS status, immune reconstitution, and opportunistic infections—poses a barrier to definitive conclusions about subgroups that may be more vulnerable to ICIs-related complications.

Response rates to treatment varied considerably, with ORR ranging from 13% to 75%. Much of this variability appears attributable to differences in treatment regimen (monotherapy vs. combination), study design, and sample size. Importantly, response rates in PWH fell within expected ranges for ICI-treated NSCLC more broadly, suggesting preserved antitumor immunity despite underlying HIV. DCR also demonstrated clinical benefit in a substantial proportion of patients.

There is conflicting data about the relationship between CD4^+^ T cell count and irAEs. Higher CD4^+^ T cell counts before receiving ICIs therapy may be linked to a higher risk of irAEs, according to some research, whereas low CD4^+^ T cell counts in participants undergoing ICIs therapy may be linked to a higher risk of irAEs [30]. Consistent with the first hypothesis, it was demonstrated in a prospective cohort study that individuals with a CD4 T cell count <200/μL at the start of ICIs have a greater risk of irAEs [29]. According to the literature, which advises stopping systemic medication in the event of grade 3–4 toxicity as well as moderate-dose to high-dose corticosteroid therapy, immunotherapy was typically stopped after the commencement of immune-mediated toxicities. About 81 people passed away during the research, resulting in a survival rate of 49.2% at 12 months and 68.6% at 6 months [48,49]. The survival rate seems to be higher for those with Hodgkin’s, cutaneous melanoma, CD4 nadir ≥100 cells/μL, and CD4:CD8 ratio ≥0.4. Since ICIs treatment did not lower the high risk of death in PWH with cancer whose CD4 count and CD4:CD8 ratio were low, Assoumou et al.’s findings support the significance of CD4 count and CD4:CD8 ratio in the prognosis of PWH with cancer [29]. This emphasizes how urgent it is to treat HIV-positive people before their immune systems suffer severe harm.

Our research is completely comforting, demonstrating that ICIs medication has no negative effects on immunological measures or HIV virological control in this cohort [21,22]. During ICIs treatment, HIV remained suppressed. In fact, during ICIs treatment, 85.1% of subjects had undetectable HIV-RNA. Furthermore, the CD4:CD8 ratio and the CD4 and CD8 numbers stayed constant. These results are predicted given that HIV medication has a significant impact on CD4 cell count and HIV VL, and that ARV treatment has remained constant over time, with only nine participants altering their baseline ARV treatment.

### Strengths and Limitations

Methodologically, most included studies were of high quality, employing valid outcome measures and clear statistical reporting. Nonetheless, several limitations were noted. First, the inclusion of retrospective cohorts and small sample sizes limits the precision of pooled estimates. Second, incomplete or inconsistent reporting of key variables, such as smoking status, autoimmune comorbidities, and prior therapies, reduces the comparability across studies. Finally, most cohorts lacked control groups, precluding direct comparisons to HIV-negative patients.

Another important limitation of the available evidence is the inconsistent reporting of HIV-related immunologic variables, including viral load, CD4/CD8 ratio, and AIDS status. This lack of uniformity restricts the ability to evaluate how baseline immune function may affect both efficacy and immune-related toxicity of ICIs in people with HIV. Moreover, without consistent virologic and immunologic data, it is difficult to identify potential thresholds of immune competence associated with better treatment outcomes or increased risk of adverse events. Future studies should therefore adopt standardized reporting of these parameters to facilitate more robust cross-study comparisons and meta-analytic evaluations.

Despite these limitations, this review provides valuable insight into an underrepresented patient population. While a meta-analysis could have provided pooled effect estimates, the included studies exhibited considerable clinical and methodological heterogeneity—including variation in study design, small and uneven sample sizes, and inconsistent reporting of efficacy and immunologic endpoints. Under such conditions, quantitative synthesis would have risked producing spurious or non-generalizable results. Thus, a descriptive synthesis was deemed methodologically sound and consistent with PRISMA guidance for evidence integration when meta-analysis is inappropriate.

The findings support the notion that ICIs are both safe and effective in PWH with NSCLC, particularly those with virologically suppressed HIV and stable immune function. However, the heterogeneity in reporting underscores the need for standardized data collection and prospective trials focused specifically on this population.

Advances in both oncology and HIV therapeutics have made it increasingly feasible to treat HIV and cancer simultaneously. Current evidence supports the continued use of combination antiretroviral therapy (cART) during ICIs treatment, as viral suppression and immune reconstitution are critical for maintaining antitumor immune responses. Preferred ART regimens in this setting typically include integrase strand transfer inhibitors (e.g., dolutegravir, bictegravir) combined with tenofovir and emtricitabine or lamivudine, owing to their favorable toxicity profiles and minimal interactions with monoclonal antibodies. Pharmacokinetic data show no clinically significant interaction between ICIs (nivolumab, pembrolizumab, atezolizumab) and these ART agents [23].

Furthermore, emerging research suggests that both ART and ICIs may act synergistically to enhance T-cell function and reduce immune exhaustion. In experimental models, HIV latency-reversing agents and PD-1 blockade have shown potential to improve viral reservoir clearance while promoting antitumor immunity. Ongoing trials are exploring combined immunomodulatory approaches, such as PD-1/PD-L1 inhibitors alongside therapeutic HIV vaccines or toll-like receptor agonists that may benefit patients living with both HIV and malignancy. Therefore, a carefully coordinated therapeutic strategy, involving infectious disease and oncology specialists, is essential to optimize efficacy and minimize overlapping toxicities in this dual disease context [50,51].

## 5. Conclusions

This systematic review demonstrates that immune checkpoint inhibitors offer a viable treatment option for people with HIV and NSCLC, yielding outcomes that are broadly consistent with those observed in HIV-negative populations. Most patients achieved disease control with manageable safety profiles, and HIV-related immunologic parameters remained stable during therapy. Importantly, the majority of patients had undetectable viral loads and maintained ART adherence throughout treatment. Future prospective studies with standardized HIV-specific reporting are needed to better define optimal ICI use in this population.

## Data Availability

No new data were created or analyzed in this study. Data sharing is not applicable to this article.
